# Exposure to exposure: A protocol for leveraging exposure principles during training to address therapist-level barriers to exposure implementation

**DOI:** 10.3389/fpsyt.2023.1096259

**Published:** 2023-02-15

**Authors:** Joshua Kemp, Kristen Benito, Jennifer Herren, Zoe Brown, Hannah E. Frank, Jennifer Freeman

**Affiliations:** ^1^Department of Psychiatry and Human Behavior, Warren Alpert Medical School, Brown University, Providence, RI, United States; ^2^Pediatric Anxiety Research Center at Bradley Hospital, Riverside, RI, United States

**Keywords:** exposure therapy, exposure process, therapist training, mechanism, implementation

## Abstract

**Background:**

Exposure therapy is a highly effective but underutilized treatment for anxiety disorders. A primary contributor to its underutilization is therapist-level negative beliefs about its safety and tolerability for patients. Given functional similarities between anxious beliefs among patients and negative beliefs among therapists, the present protocol describes how exposure principles can be leveraged during training to target and reduce therapist negative beliefs.

**Methods:**

The study will take place in two phases. First, is a case-series analysis to fine-tune training procedures that is already complete, and the second is an ongoing randomized trial that tests the novel exposure to exposure (E2E) training condition against a passive didactic approach. A precision implementation framework will be applied to evaluate the mechanism(s) by which training influences aspects of therapist delivery following training.

**Anticipated results:**

It is hypothesized that the E2E training condition will produce greater reductions in therapists’ negative beliefs about exposure during training relative to the didactic condition, and that greater reduction in negative beliefs will be associated with higher quality exposure delivery as measured by coding of videotaped delivery with actual patients.

**Conclusion:**

Implementation challenges encountered to date are discussed along with recommendations for future training interventions. Considerations for expansion of the E2E training approach are also discussed within the context of parallel treatment and training processes that may be tested in future training trials.

## Introduction

Anxiety disorders are among the most common early mental health problems with prevalence rates in the range of 20% for children and young adults (1). If left untreated, anxiety disorders in childhood follow an unremitting pattern that leads to a cascade of developmental consequences and costly adult disability (2, 3). Exposure-based cognitive-behavioral therapy (CBT) is a highly efficacious psychosocial treatment for child anxiety disorders (4). Although CBT for anxiety may consist of several components, there is consensus among experts that the most effective ingredient in CBT for anxiety is exposure (5–7). Evidence also supports exposure as the most effective treatment component for adult anxiety disorders [e.g., (8)]; thus, exposure therapy is the treatment of choice for anxiety disorders across the lifespan, making it one of the most broadly applicable and essential components of evidence-based practice (EBP).

Despite clear efficacy, exposure is one of the least utilized EBPs to treat anxiety or OCD. As few as 7% of therapists and patients report providing or receiving exposure therapy (9–12). Furthermore, when exposure is utilized, it is often delivered in a manner that differs markedly from the prolonged and intense approach recommended in treatment manuals (13, 14). Although there are multiple potential strategies for reducing barriers to clinician delivery of EBPs, one logical remedy would be to increase the number of clinicians trained in exposure therapy, as only 12–28% of providers have received training to deliver this treatment (9). However, even among providers with specialized training in exposure, less than half report they have ever used it with appropriate patients (9), and exposure is associated with disproportionately low rates of therapist adherence and competence (15). This suggests current training approaches for exposure therapy are in need of innovative and targeted approaches capable of addressing known barriers to its dissemination and quality delivery.

Effective therapist training is a core component for the dissemination and implementation of EBPs (16). The current “gold standard” for training includes a workshop, accompanying manual, and clinical supervision (16, 17). Although there is consensus that supervision, consultation, and/or feedback about performance is necessary (18), even the highest doses of these training approaches have not been sufficient to produce optimal outcomes. For example, in one study randomizing therapists to different “doses” of training, only 54% of clinicians receiving the highest “dose” reached proficiency levels (17). This is concerning given that training is unlikely to be effective if therapists cannot achieve a level of proficiency that would allow them to feel comfortable incorporating the new approach into their typical practice (19). Implementation of EBPs can be improved by understanding the *mechanisms* through which therapist training produces a change in practice behavior. Existing implementation research is limited by a lack of emphasis on assessing implementation mechanisms (20), or how and why implementation strategies such as training operate to lead to change. Thus, implementation of EBPs is likely to be improved by assessing mechanisms that lead therapist training to produce a change in practice behavior.

### Barriers associated with exposure underutilization and delivery quality

Exposure therapy suffers from a public relations problem (21), and therapists along with many individuals in the public, tend to think of exposure as “flooding” [i.e., making people do things that are too difficult and very overwhelming; (22)]. Research indicates therapists’ negative beliefs about the dangerousness and intolerability of exposure therapy for patients and themselves is a primary barrier to their utilization and optimal delivery of the treatment (23–25). To measure therapist negative beliefs about exposure, Deacon et al. (23) developed the Therapist Beliefs about Exposure Scale (TBES) and validated the measure in a diverse and nationally representative sample of practitioners (N = 637). Providers completed the TBES along with case vignettes depicting the use of exposure therapy with anxious patients. The vignettes presented four patients with four different anxiety disorders (social anxiety disorder, panic disorder, post-traumatic stress disorder, and obsessive–compulsive disorder). Therapists rated their level of concern with: (a) patient difficulty tolerating the exposure task, (b) ethicality of the exposure task, (c) therapist discomfort, (d) negative effects on the therapeutic relationship, (e) risk of harming the patient, (f) necessity of the task for an optimal outcome, and (g) personal willingness to provide the depicted exposure. The TBES exhibited acceptable psychometric properties, and higher levels of negative beliefs were strongly associated with more negative reactions to the depiction of exposure in the four case vignettes, indicating good construct validity.

### Relationship between negative beliefs and exposure utilization and delivery quality

Research supports the link between negative beliefs about exposure and its underutilization among therapists. A survey by van Minnen et al. (26) found that most self-identified trauma experts did not use exposure therapy with patients, and that underutilization was related to negative beliefs (i.e., fears of patient dropout). Further, other researchers have also noted that therapists’ decision not to use exposure therapy is related to beliefs about its dangerousness or potential for negative events, such as symptom exacerbation (27), patient decompensation (28), direct patient harm (21), and eventual treatment dropout (26). In addition, therapists elect not to use exposure due to worries about it being too aversive for their patients (29, 30), or a perceived increased likelihood of negative events for the therapist such as risk of malpractice lawsuits or eliciting intolerable affect in the therapist while the patient completes exposures (31, 32). However, serious negative consequences as a result of delivering exposure therapy are incredibly rare (33). While negative beliefs about exposure can include concerns about treatment utility (e.g., “it will not work”), it is far more common for negative beliefs to center on anxiety-based perceptions of the dangerousness or intolerability of exposure activities for the patient and/or therapist. All references to negative beliefs hereafter will pertain to anxiety-based reluctance to utilize and optimally deliver exposure therapy.

Negative beliefs about exposure have also been associated with its suboptimal delivery, which is characterized by patterns of delivery behavior that deviate from the prolonged and intense manner advocated by exposure theorists and treatment manuals (34). In a national survey of exposure therapists who reported using interoceptive exposure to treat panic disorder, approximately 40 % of therapists prescribed controlled breathing strategies during exposure--70 % of whom did so in order to make exposure exercises less aversive and more acceptable (13, 35). In another national survey, responses to an OCD exposure vignette revealed that negative beliefs were associated with an increased likelihood of emphasizing distress reduction techniques and allowing for the use of safety behaviors (i.e., forms of avoidance). These findings are consistent with those of Harned, Dimeff et al. (36) demonstrating that negative attitudes toward exposure predicted a number of aspects of cautious exposure delivery, including mishandling of patient avoidance, providing patients with reassurance, and the premature termination of exposure tasks. Given the well-documented role of negative beliefs in stifling the dissemination and optimal delivery of exposure therapy, it is clear that successful training interventions must develop targeted strategies for addressing this unique barrier.

**Figure 1:**
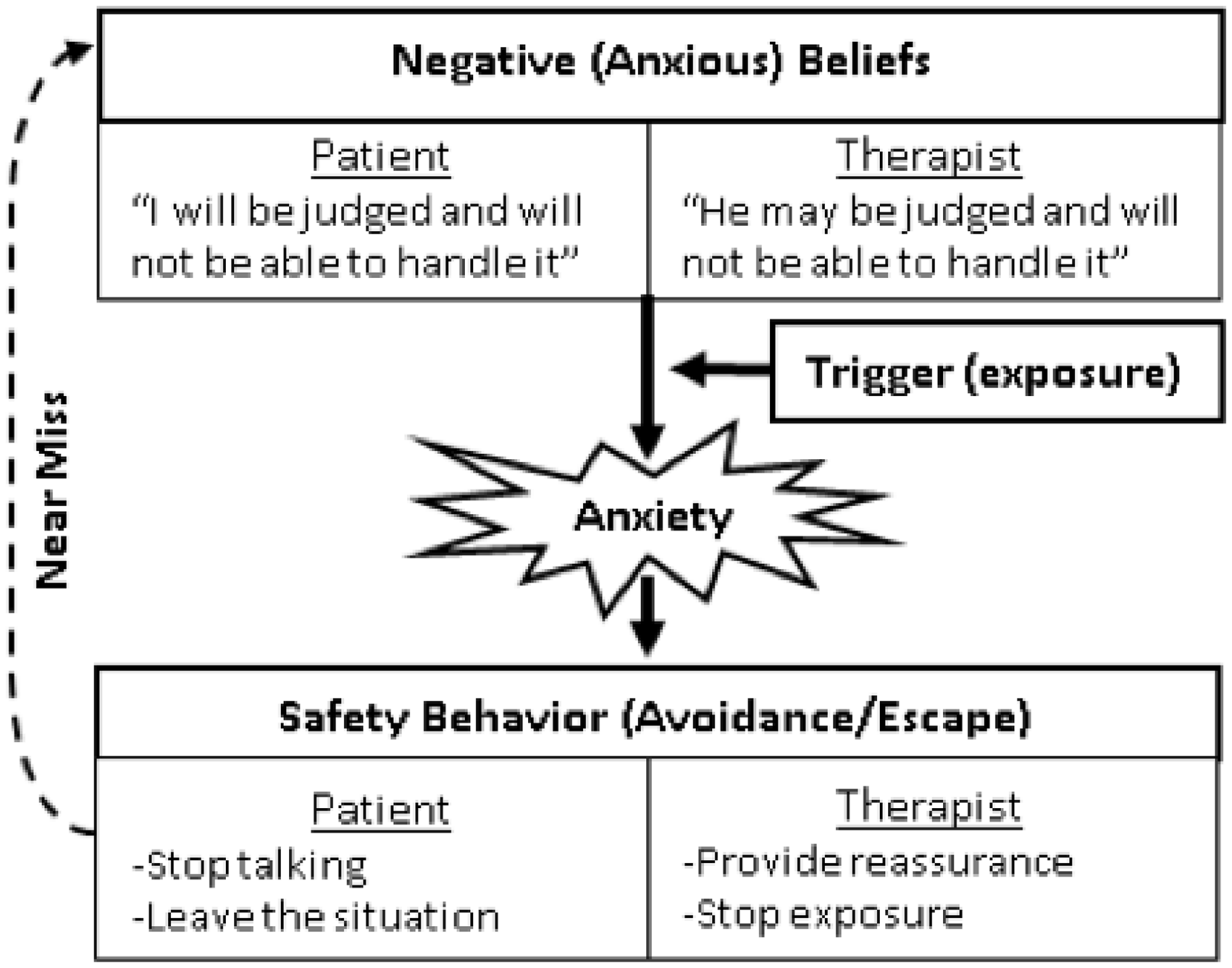
CBT model of patient and therapist anxiety.

Research on training as an approach to address negative beliefs has demonstrated the promise of didactic workshops. Workshops can reduce negative beliefs during training by addressing common myths and knowledge gaps about how exposure is delivered, and by emphasizing that it is meant to be a collaborative and gradual process. In a sample of 162 community therapists (mostly masters’-level and exposure naïve), Deacon et al. (23) measured negative beliefs before and after a day-long workshop focused on improving knowledge about exposure therapy. Beliefs about exposure measured before and after the training revealed that negative beliefs were significantly reduced following the didactic training, *p* < 0.001, *d* = 1.50. Although this appears promising, studies of therapist training approaches indicate that didactic training is generally insufficient for changing therapist behavior (16, 37), and after didactic training therapists often continue to underutilize and suboptimally deliver exposure. Importantly, continued use of suboptimal delivery behaviors after didactic training appears to be driven by remaining negative beliefs after didactic training (23, 38), suggesting that didactic approaches are not a potent ‘lever’ for sufficiently reducing negative beliefs. Evidence supports experiential training strategies as drivers of therapist behavior change, but more research is needed to understand the mechanisms by which experiential tasks promote the subsequent implementation of a given EBP (39). Exposure therapy is built upon a strong rationale (i.e., CBT model of anxiety; Figure 1) and a well-established set of cognitive and behavioral change mechanisms (40–42), and it stands to reason that the same exposure procedures and mechanisms that augment patient anxiety may also prove successful in reducing therapists’ anxiety-based negative beliefs during training.

### Applying the CBT model of anxiety to patients *and* therapists

The cognitive-behavioral (CBT) model of anxiety disorders is a well-researched framework for conceptualizing the process by which patient anxious beliefs and avoidance behaviors are maintained. A pared down version of the CBT model is presented in Figure 1 to illustrate its application to both patients and their therapists in the context of conducting exposures. Patients. At the top of the model are *negative beliefs* about feared situations. When these beliefs are “*triggered*” by the presence of a feared stimulus, the individual becomes concerned about negative outcomes and engages in *safety behaviors* – actions intended to prevent or reduce negative outcomes. Although seemingly helpful in the moment, research suggests safety behaviors maintain negative beliefs by allowing a person to avoid or escape the situation, which then prevents the patient from learning whether anything bad would have actually happened if they had not used a safety behavior (43, 44). This creates a “*near miss*” feeling that reinforces negative beliefs (34). The rationale for exposure therapy is to gradually approach feared situations, without using safety behaviors, to learn that the situation is less dangerous and intolerable than predicted.

Therapist negative beliefs about exposure closely resemble the anxious beliefs endorsed by their patients. As proposed by Becker-Haimes et al. (45), clinician maladaptive anxious avoidance is likely to function similar to patients’ anxiety. Specifically, when interventions such as exposure elicit clinician anxiety (e.g., about causing harm to patients), it may result in clinician avoidance of delivering the intervention, which in turn will relieve anxiety and reinforce avoidance of delivering the intervention. As illustrated in Figure 1, therapists often fear the occurrence of the same negative outcomes as their patients when engaging in exposure therapy. Just as patients use safety behaviors to mitigate perceived danger, therapists too use safety behaviors to prevent feared outcomes. Therapists with negative beliefs about exposure therapy rely on safety behaviors to reduce patient distress (e.g., providing reassurance, minimizing the intensity of exposure), avoid negative patient outcomes (e.g., permitting the use of safety behaviors, terminating exposure tasks prematurely), reduce the therapist’s own discomfort during treatment (e.g., only assigning exposure tasks as homework, failing to model exposures for the patient), and control patient perceptions of the therapist (e.g., apologizing for distress evoked by exposure). Delivering exposure therapy in this cautious manner is problematic because it deviates from the widely advocated prolonged and intense delivery thought to optimize outcomes (34). Thus, cautious delivery behaviors may undermine the effectiveness of exposure and serve as erroneous confirmatory evidence for therapists harboring negative beliefs about its use in their practice. Therapists engaged in safety behaviors are also subject to the same “near-miss” maintenance process as their patients, and may misconstrue the non-occurrence of negative exposure outcomes to the use of safety behaviors and perpetuate both negative beliefs and suboptimal delivery behavior.

### Conceptualizing exposure training as “exposure to exposure”

To the extent that patient fears and therapist negative beliefs are maintained by a similar CBT model of anxiety maintenance, it stands to reason that the same exposure procedures shown to be effective for patients may also be leveraged in therapist training interventions to reduce negative beliefs and promote optimal delivery behavior (i.e., conduct training as “exposure to exposure”). For example, exposures for therapists could involve repeated trials of the same exposure tasks they might expect to prescribe for a patient, and continuing until they feel adequately confident in the safety and tolerability of the tasks--even when exposure involves highly anxiety-provoking items. A recent study of strategies for targeting negative beliefs using training techniques derived from social-cognitive learning theory, found that behavioral strategies (i.e., self-exposure) is more effective at reducing negative beliefs than a standard didactic training (39). These findings provide initial evidence that training can be tailored to target and reduce negative beliefs; however, research has yet to determine whether this leads to a change in practice behavior.

### Applying a mechanistic framework to the design and testing of training interventions

**Figure 2:**
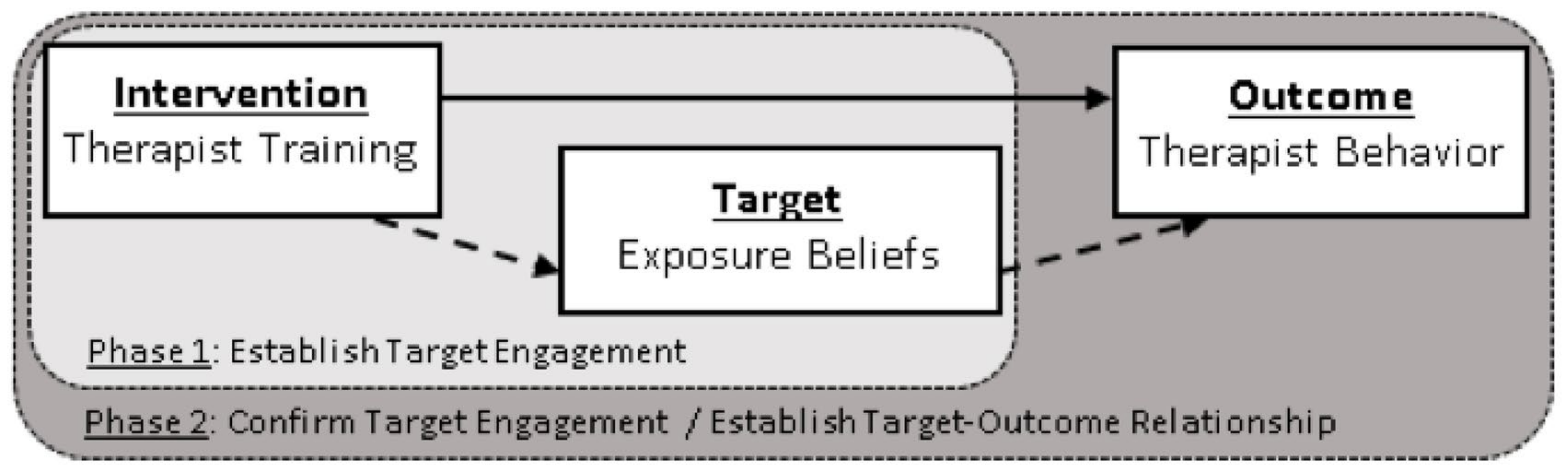
Experimental therapeutics framework.

Experimental therapeutics is a mechanism-testing framework promoted by the National Institute of Health (NIH) Science of Behavior Change (46) and the National Institute of Mental Health [NIMH; (47, 48)] to support the rigorous evaluation of candidate change mechanisms as a more efficient means to intervention development. Perhaps because of its roots with novel biological interventions and targets, it has been applied infrequently in the development of novel service interventions, despite explicit calls to do so (48, 49). The experimental therapeutics framework goes beyond asking whether or not an intervention works to clarify how and why the intervention works. The broad goals of this approach are: (1) to select a target mechanism with preliminary evidence demonstrating malleability to the proposed intervention, and (2) to demonstrate that change in the target also affects planned outcomes. More rigorous applications of experimental therapeutics should aim to demonstrate a causal dose–response relationship between the intervention, target mechanism, and planned outcomes. As indicated in Figure 2, the progression of the present investigation is designed to fit the experimental therapeutics model.

A recently proposed adaptation of the experimental therapeutics framework, termed *precision implementation*, provides guidelines for advancing the application of experimental therapeutics along the translational spectrum for use in evaluating dissemination and implementation efforts, including training interventions (50). Given the multi-level factors (i.e., individual, organization, and environmental) that influence implementation outcomes, it is important for a framework to guide the appropriate selection of an intervention, mechanism, and outcome that are aligned on the same level of analysis. For instance, an intervention or implementation strategy intended to change provider behavior (e.g., training) should aim to engage a provider-level mechanism (e.g., negative beliefs) and lead to a change in a provider-level outcome (e.g., utilization of exposure with anxious patients). Unfortunately, little is known about the mechanisms through which components of therapist training exert their effectiveness. Without this knowledge, approaches to improve training might be imprecise and over-reliant on burdensome strategies that do not match the needs of practice settings (e.g., increased training and supervision hours). The present study provides a case example of how experimental therapeutics, and more specifically a *precision implementation* approach can be used to efficiently evaluate mechanism engagement within a novel training intervention.

### Current study

The current protocol paper describes novel procedures for leveraging exposure therapy principles to design targeted training strategies for reducing therapist negative beliefs about exposure. The study enrolled therapists providing child mental health services in patients’ homes or community. Home-based services are utilized by some of the most impaired pediatric mental health patients, yet providers in this setting typically have less experience than their hospital-based counterparts. A recent survey of home-based providers suggests a majority have less than 5 years of field experience, and over 80% of these providers report no available coursework or supervision specific to the delivery of home-based services (51). Research indicates negative beliefs about exposure are higher among providers working with younger patients, greater levels of comorbid conditions, the absence of specialized training in the treatment of anxiety disorders, and less graduate education (23, 25). Thus, many of the known predictors of negative beliefs about exposure are embodied by home-based therapists and the patient population they treat. Selecting a sample with particularly high levels of the intervention target is a strength of the current design.

Evaluation of the exposure to exposure (E2E) training intervention took place in two phases. The first phase was a case-series analysis (*N* = 6 therapists) intended to fine-tune training procedures and establish the necessary dose of experiential training for therapists’ negative belief levels to reach an *a-priori* benchmark. The second phase was a randomized trial (*N* = 36 therapists) testing whether the targeted E2E training approach, relative to didactics alone, was a more potent intervention for reducing negative beliefs. Specifically, it was hypothesized that therapists in the E2E training condition would evidence significantly lower negative beliefs about exposure relative to the didactic condition at post-workshop. As an added layer of rigor, levels of general beliefs about EBP were measured along with negative beliefs at pre-, mid-, and post-workshop to demonstrate the specificity of the E2E condition’s effect on the planned target (i.e., negative beliefs) while exhibiting comparable levels of EBP belief change relative to the didactic condition. It was also hypothesized that negative belief change during the workshop training would be associated with therapist delivery behavior when implementing exposure with anxious patients in the consultation phase of the study, such that greater reduction in negative beliefs would be associated with: (1) a higher rate of optimal delivery behaviors, (2) a lower rate of suboptimal delivery behaviors as measured by both self-report and observational coding data, and (3) a higher rate of exposure utilization across sessions. A detailed accounting of study methods, practical adjustments, and other implementation considerations are presented below.

## Methods

### Participants

#### Therapist participants

Therapists were recruited from in-home service teams at two community mental health agencies in southeast Massachusetts and Providence, RI. A total of 42 in-home providers completed the workshop training across Phase 1 (*N* = 6) and Phase 2 (*N* = 36) of the study.

#### Patient participants (phase 2 only)

As of the writing of this paper, we have recruited 21 patient participants in the Phase 2 training trial. Eligible participants included children and adolescents ages 5–17 years seeking treatment for an anxiety disorder. Patient participants needed to be existing patients at one of our partnering sites, and were referred by their study-trained therapist if they presented with primary or co-primary diagnosis of Separation Anxiety Disorder, Specific Phobia, Social Anxiety Disorder, Panic Disorder, or OCD or GAD using a semi-structured interview. Patient participants could present with a wide range of comorbid conditions so long as anxiety was primary or co-primary, with the exception of delusional disorders, pervasive developmental disorders, conduct disorder, or acute suicidality (i.e., current plan or intent) as such symptoms may significantly impair engagement with exposure tasks or require a focus on other immediate services. Exclusion criteria were designed to be minimally restrictive and reflect as closely as possible the patient populations served by the partnering home-based clinics.

#### Training and treatment sites

Our primary partner (Agency 1) was a large community mental health agency (CMHA) in Massachusetts, serving over 5,000 children and adults each year in their outpatient clinics, and employing nearly 200 therapists (>95% masters-level) and supervisors across six sites. Home-based services are a specific offering within the agency’s outpatient department, but it is important to note that services provided by the home-based providers enrolled in this study took place in patients’ homes or community, whichever was most relevant for treating their symptoms. At the outset of the project, Agency 1 provided in-home services at four locations split across two different service divisions. All treatment services were conducted at the participating CMHA outpatient sites and clinical care was delivered in accordance with agency policies. Patient recruitment challenges stemming from therapist retention issues at Agency 1 during the Phase 2 training trial led to the addition of a second CMHA (Agency 2) based in Rhode Island. Agency 2 was a single clinical site with approximately 20 therapists providing in-home psychotherapy throughout Rhode Island.

#### Referral and recruitment processes

Therapists were given instructions about how to identify appropriate study cases and the process for making referrals during the workshop training. Patients were only screened for study eligibility by study staff after being assigned to a study-trained therapist. Therapists discussed the study with patients on their caseload that presented with relevant anxiety concerns, and if interested, therapists completed an online Consent to Contact form that allowed the study team to reach out to their patient. Screening was a multi-gate process intended to reduce burden on patients. First, patients completed a 30-min phone screen with a study research assistant. The phone screen was conferenced with a study investigator (JK, JH, or JF), to determine initial eligibility, before scheduling a full diagnostic interview (90-min) to determine final eligibility.

### Measures

#### Therapist workshop measures

Therapist negative Beliefs about Exposure Scale [TBES; (23)]. The TBES assesses the extent to which therapists endorse 21 negative beliefs about exposure therapy. Participants use a 5-point scale (0 = “disagree strongly” to 4 = “agree strongly”) to indicate the extent to which they agree with such items as “most clients have difficulty tolerating the distress exposure therapy evokes.” The TBES is the primary outcome measure for assessing target engagement. Evidence Based Practice Attitudes Scale [EBPAS; (52)]. This is a brief (15-item) measure that assesses four general attitudes toward adoption of EBPs, including such items as “research based treatments are not clinically useful.” This measure will be used to demonstrate the specificity of target engagement with the E2E approach. Specificity is exhibited by the extent to which the training groups differ on belief change while remaining equivalent in attitudes about EBP in general. Exposure Knowledge (36). We have condensed the original 49 multi-choice item measure by selecting 12 multiple-choice items that best fit the didactic content of our training, for instance, “Why is it important to block avoidance during exposure tasks?” Exposure Self-Efficacy (36). This is a 27-item measure of therapists’ confidence in delivering exposure therapy. All items began with “I feel confident in my ability to,” and an example item is “Conduct imaginal exposure.” Items are rated on a 5-point Likert scale ranging from 1 (not confident) to 5 (very confident). This measure has demonstrated high internal consistency and predictive validity in determining the frequency of self-reported clinical use of exposure therapy. Beliefs about Exposure Scale-Training (BES-T). This is a beliefs process measure designed for this study to measure belief change during training activities. The scale consists of six items that assess therapists’ reservations about the safety and tolerability of exposure therapy. The first five items are rated on a 0 (“Completely Disagree”) to 100 (“Completely Agree”) scale and assess perceptions of safety and tolerability of exposure for their patients as well as themselves. The sixth item stated, “I feel very confident in my ability to deliver exposure therapy and I am committed to using it with my anxious clients,” and the response options were either “Not Yet” or “No more practice – I am ready.” Therapists continued with experiential tasks until they endorsed the “I am ready” item. This item is a go/no-go item intended to solicit self-perceptions of whether therapists felt ready and able to use exposure therapy patients with anxiety. Training Acceptability Rating Scale [TARS; (53)]. The TARS is a measure of therapists’ satisfaction with trainings that has good psychometric properties. Participants rate training acceptability and perceived utility on Likert scales (total scores = 6–63), in addition to qualitative feedback.

#### Treatment delivery and consultation measures

Exposure Guide (EG). The EG is a quality monitoring tool that has been validated in previous and ongoing exposure therapist training studies (54, 55). The tool is completed by therapists following a treatment session, and rates the extent to which specific behaviors occurred during the session, along with quality indicators such as: using a hierarchy to choose the exposure, patient participation in exposure selection, clear presentation of exposure task, taking anxiety ratings, post-processing the exposure, discussing avoidance after the session, gathering new information about anxiety, tapping the core fear, and whether the exposure was too hard or too easy. The EG was designed to assess features of exposure therapy that have been shown to predict outcome (54, 56, 57). Exposure Process Coding System (EPCS) (56). The EPCS is a “gold-standard” measure of exposure delivery quality and will be applied to therapists’ audio and videotaped treatment sessions with study patients in phase two of the study. The EPCS is a microanalytic, time-stamped coding system that uses Noldus Observer software to capture process variables as they occur during exposures. EPCS codes measure therapist, patient, and parent (if present) behaviors or statements during an exposure. The EPCS also includes several measures of habituation: subjective units of distress (SUDS), coder-rated anxiety level, and coder-rated habituation. Unlike the EG which relies on global impressions of session content using clinical judgement, EPCS is a more strict “event by event” system for recording therapist behaviors. EPCS demonstrates strong psychometric properties, including inter-coder reliability (with Bachelor’s-level raters), construct validity, and predictive validity (54, 56, 57). EPCS Coders and Training. A BA-level research assistant will code audio and videotaped session data, and a masked co-investigator (KB) will code 10% of videotaped session data to calculate inter-coder reliability. Training includes group discussions, practice coding with feedback, and practice coding to criterion. The rater will attain reliability on the practice tapes (K > 0.80) before coding study tapes. If coder drift is detected, re-training will occur and remaining study tapes will not be coded until the reliability criterion is re-established.

#### Measurement considerations

**Figure 3:**
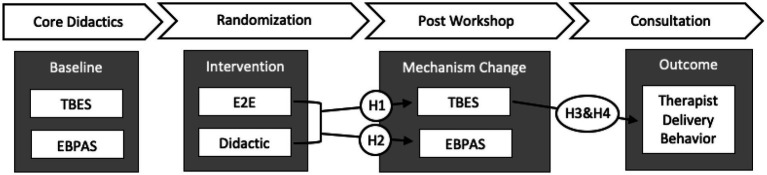
Training activities, hypotheses, and measurement strategy. EBPAS = Evidence Based Practice Attitudes Scale; TBES = Therapist negative Beliefs about Exposure.

While piloting measure administration during the case-series in Phase 1 it became clear that repeatedly administering the TBES after rounds of the experiential tasks was burdensome, interrupted the learning flow, and posed a potential risk of sampling error. This led to the development of the brief BES-T measure to allow for frequent monitoring of change in the belief target without interrupting training and potentially undermining the potency of experiential learning. During the case-series analysis, therapists who endorsed feeling ready to use exposure on the BES-T go/no-go item (4 of 6 therapists) following E2E procedures also reported post-workshop TBES scores below the *a-priori* criterion score, while those who endorsed not yet feeling ready to use exposure (2 of 6 therapists) were above the TBES criterion score at post-workshop. It is possible therapists could simply endorse feeling ready to use exposure to end experiential training tasks, but the consistency between endorsement of this item with our TBES clinical criterion score would suggest therapists answered truthfully in the Phase 1 case-series. Future measurement strategies will take into closer consideration the possibility of therapists potentially endorsing a willingness to use exposure in order to discontinue experiential activities. The sixth item in the BES-T measure is also a double-barreled question assessing both therapists’ confidence and intent to use exposure that will likely need to be broken out into two separate questions in a future test of the E2E training intervention. At present, it is possible a therapist could be confident but still not intend to use exposure and the current question configuration would not allow sensitivity to this possibility. Resulting finding will be interpreted in light of this consideration. Time points for sampling therapist beliefs were carefully chosen to allow for mechanism evaluation. As depicted in Figure 3, the intervention target was assessed before, during, and after the experimental E2E training strategies were implemented. An easy to administer process measure for assessing incremental change in the belief target was also implemented during experiential E2E training tasks to measure dose response, and a rigorous micro-analytic coding system was used to quantify the outcome of interest (i.e., therapist delivery behavior; Table 1).

**Table 1:** Measurement strategy and timepoints.

Measure (*Objective*)	Timepoint					
	Baseline	End of core didactics	During experiential (E2E only)	End of workshop	Consult	End of study
TBES (*Primary target*)	X	X		X		X
EBPAS (*Target specificity*)	X	X		X		X
Knowledge (*Candidate target*)	X	X		X		X
Self-efficacy (*Candidate target*)	X	X		X		X
BES-T (*Target process*)			X			
Training satisfaction (*QI*)				X		X
EPCS (*Outcome*)					X	

BES-T = Beliefs about Exposure Scale – Training; EBPAS = Evidence Based Practice Attitudes Scale; EPCS = Exposure Process Coding System; Knowledge = Exposure Knowledge Measure; Self-Efficacy = Measure of Exposure Delivery Self-efficacy; TBES = Therapist negative Beliefs about Exposure; Training Satisfaction = Training Acceptability Rating Scale (TARS).

### Study procedures

#### Phase 1: Case series (complete)

Six therapists from two different clinical sites were recruited for a case-series designed to establish target engagement and determine optimal procedures for conducting the behavioral training strategies in Phase 2. During the case-series, therapists first received an 8-h didactic training on exposure therapy and the principles of its delivery to orient them to the treatment approach. Following didactics, therapists completed a series of behavioral training strategies that include both self-exposures and partner exposures. This information was used to ensure that behavioral strategies in Phase 2 of the study were appropriately dosed to engage negative beliefs, consistent with the precision implementation approach.

#### E2E design considerations

We considered a wide range of behavioral strategies with the potential to augment negative beliefs, including: delivery with confederate patients, watching exposure session videos, imaginal exposure, or exposures with the trainer in the therapist or patient role. Ultimately, we selected the two behavioral strategies (self-and partner-exposures) for this study based on preliminary support in the training literature (38, 39), our experiences with the effectiveness and tolerability of implementing these behavior strategies during training, and their face-valid match with the four main domains of therapist negative beliefs (i.e., Exposure is intolerable (1) for my patients and (2) for me; Exposure is dangerous (3) for my patients and (4) for me). We also selected these strategies based on their ability to be transportable and therefore lend to the dissemination and scalability of the training.

#### Optimizing E2E during experiential training tasks

Principles of exposure delivery for patients include: generating a fear hierarchy, selecting and titrating appropriate exposure tasks, engaging threat perceptions during exposure, tracking change in anxiety and anxious beliefs, and consolidating learning.

##### Generating a fear hierarchy

Therapists were presented with three partially complete fear hierarchies pertaining to social, contamination, and panic concerns. The goal was to represent three diverse fear domains that may elicit at least some anxiety for therapist participants. Each hierarchy consisted of seven representative exposure tasks ranging from easy (e.g., “touch snack to tabletop and eat it”) to more challenging (e.g., “touch snack to inside of garbage can and eat it”). Therapists reviewed each hierarchy and selected the one that had tasks they thought were most relevant to their own worries. Next, they added three more personally relevant exposure tasks (low, moderate, and high anxiety) to the hierarchy to practice generating unique exposure scenarios; this resulted in a personalized fear hierarchy for each participant, with a range of exposure ideas for them to choose from when initiating their first round of self-exposures. Our experience in this and in other studies suggests that all therapists have been able to identify “challenging but doable” exposure tasks using this approach and titration methods as described below.

##### Selecting and titrating appropriate exposure tasks

After individualizing their fear hierarchies, therapists were asked to select a starting task from the list that felt like a “challenging but doable” scenario. The trainer emphasized the importance of selecting an item that brings up at least some anxiety in order for therapists to experience the true process of exposure for themselves. After the first self-exposure trial, therapists were given the option to repeat the same exposure or modify the challenge level to be harder or easier in their second round of self-exposure. After completing a second self-exposure, therapists partnered up and took turns playing the role of patient or therapist. Those in the patient role discussed the recent self-exposure they had conducted and those in the therapist role helped their partner select and titrate their next exposure task.

##### Conducting exposures in a prolonged and intense manner

Before engaging in each exposure task, therapists were instructed to persist with their exposure task past the point at which they assumed a negative outcome would occur or past the point at which they would be inclined to stop; these instructions align with theories of optimal exposure delivery for promoting new learning about the dangerousness and tolerability of their feared situation (58, 59). Therapists were given up to 5 min to complete each round of self-exposure and 8 min to complete each partner exposure; these durations were selected to allow enough time for individuals to thoroughly approach their feared item/situation while also keeping the exposure window brief enough to allow for repeated trials. Therapists were guided to select exposure tasks that could be completed within the window and allow enough time for safety learning and habituation to occur (e.g., singing to the group). This was plenty of time for most participants to fully engage with feared content and experience new safety learning.

##### Consolidating learning

Before each self-exposure task, therapists discussed which task they were attempting, their anticipatory anxiety level, and the feared outcome they were testing. Then, after completing their self-exposure, they returned to the group and reported their overall experience during the exposure and their revised estimates of the dangerousness and/or intolerability of their exposure task. A similar process took place during partner exposures for the individual in the patient role, except that those in the therapist role also reported on their experience, anxiety levels, and what they learned while directing someone else’s exposure experience. The goal of the pre-and post-processing was to practice this important element of actual exposure delivery and to witness the accumulation of new safety learning among all group members in both the patient and provider roles.

#### Phase 2: Randomized training trial (ongoing)

Therapists were randomly assigned to either a didactic-only or a didactic plus E2E training workshop which was 12 h in total. All therapists completed the same 8-h “Core Workshop,” which was followed by a condition-specific 4-h training (Didactic or E2E). Following the workshop training, therapists began using exposure therapy with patients presenting with anxiety disorders and received ongoing weekly consultation. Each therapist was encouraged to refer 1–2 patients to the study to receive ongoing consultation regarding the use of exposure therapy for patients who elected to participate in the study. In line with the gold-standard structure for therapist training, therapists with enrolled patients attended weekly, hour-long consultation conducted in a group format *via* video teleconferencing.

##### Core workshop

This was a didactic training that covered foundational information about anxiety disorders, maintenance theories and the principles of exposure delivery, including: (a) a diagnostic overview of common anxiety disorders including OCD, (b) developmental considerations, (c) presentation of the CBT model of anxiety, (d) a functional explanation of anxiety symptoms and their maintenance, (e) an introduction to four main components of exposure therapy (psychoeducation, hierarchy development, exposure tasks, and relapse prevention), (f) an in-depth discussion of the procedures for conducting each component of exposure therapy, (g) advice for troubleshooting common issues when delivering exposure therapy, (h) and a discussion of considerations for delivering exposure therapy in the home setting.

##### Didactic condition

Those in the didactic condition reviewed key concepts from the prior 8-h training, including the CBT model of anxiety maintenance, the rationale for how exposure directly counteracts the maintenance model, and principles for conducting optimal exposure tasks. Therapists in this condition also spent extra time reflecting on their own caseloads and discussing current (or past) patients that might be a good fit for exposure to assist with the process of identifying eligible patient participants.

##### Exposure to exposure (E2E) condition

Those in the E2E training condition briefly reviewed previous didactic content (1 h), before engaging in experiential training strategies. Experiential activities followed the steps of (1) generating a fear hierarchy, (2) selecting and titrating appropriate exposure tasks, (3) conducting exposure in a prolonged and intense manner, and (4) consolidating learning as described in the “Optimizing E2E” section above. After completing two rounds of self-exposure all therapists completed the BES-T. Regardless of whether they selected “No More Practice – I am Ready” on the BES-T go/no-go item, all therapists then completed two rounds of partner exposures. After two rounds of partner exposures they again completed the BES-T, and those who endorsed “No More Practice – I am Ready” were placed in a waiting room to discuss their current patient caseload and think about potential patient referrals to the study, while those endorsing “Not Yet” completed another round of partner exposure with a study confederate (research assistant) in either the patient or provider role depending on which they thought would be most helpful for increasing their exposure confidence; in all instances, those requiring additional practice elected to be in the therapist role. Therapists repeated the partner exposure task and completed the BES-T after each trial until they selected “No More Practice – I am Ready.”

##### Supervision

Following the initial workshop, therapists delivered exposure therapy with patients recruited to the study and attended weekly (60-min), condition-specific consultation meetings. All supervision took place remotely *via* Zoom. The general format of supervision included: (1) triage of any urgent patient matters, (2) allocation of extra time for therapists starting a new exposure case, and (3) rotation of the order in which therapists presented their cases to ensure equal time and focus. Supervision in the didactic condition focused on principles and procedures of exposure delivery, including troubleshooting of exposure delivery barriers, but avoided conversation about therapist anxiety and the E2E concept. Supervision in the E2E condition had a similar focus on delivery principles and troubleshooting but also prioritized each therapists’ processing of negative beliefs. Specifically, therapists were asked what negative beliefs they planned to test in their upcoming exposure sessions and they were asked to reflect on what they learned about their negative beliefs in the subsequent supervision session. Supervision in the E2E condition was the same duration and frequency as the didactic condition, as additional discussion of negative beliefs required minimal extra time or focus when embedded within the larger conversation. All supervision sessions were audio recorded to allow for fidelity monitoring.

#### Study treatment

Anxiety treatment was informed by a flexible treatment manual used by our research team in previous exposure training studies (54, 55), as well as treatment strategies employed as usual practice. The study explicitly did not require use of a specific treatment protocol, but instead encouraged “flexibility within fidelity” to the provided manual by applying a workshop knowledge about exposure principles and their intended function on patients’ presenting concerns (60). Rather, therapists were oriented to a flexible manual that outlined the basic ‘steps’ needed to support exposure, but left room for therapists to independently apply the exposure principles detailed during the workshop. Length of treatment and timing of treatment sessions (e.g., daily, every other day) was variable depending on usual care at the organization (i.e., some insurances require more face-to-face hours than others for reimbursement of home-based service packages). End of treatment as part of study participation was triggered by one of the following: (1) formal treatment termination with therapist, (2) patient no longer interested in participating in the research study, or (3) patient and therapist agree that anxiety is no longer the primary focus of treatment and switch to a new target for which exposure is not the primary treatment.

## Anticipated results

The primary goals of this study are to first establish and then confirm engagement of the negative beliefs target with the novel E2E training approach, and to also demonstrate a relationship between change in the beliefs target and therapists’ in-session delivery behaviors. Target engagement was established during the Phase 1 trial. The Phase 2 training trial will confirm target engagement by demonstrating significantly greater change in belief scores following workshop training for therapists assigned to E2E relative to the didactic group (Hypothesis 1). To test this effect, group status (E2E vs. didactic) will be entered as a predictor of post-workshop total TBES score while including a random intercept for TBES baseline score. It is anticipated that as TBES-measured belief levels approach zero, the distribution will become positively skewed. This is problematic given the goal to target and reduce belief levels as close to zero as possible. To account for the positive skew of TBES scores approaching zero, binomial-based modeling will be used to conduct a logistic regression to assess for a difference in belief reduction between those in the didactic and E2E groups following the workshop training. Figure 3 illustrates the measurement time points and pathways for testing planned hypotheses.

It is also expected that the E2E intervention will produce a specific change in negative beliefs (measured pre-and post-workshop) about exposure but not differentially affect broader beliefs about EBPs (EBPAS) in general (Hypothesis 2). Target specificity will be demonstrated by significantly greater levels of negative belief change but comparable levels of EBP belief change in the E2E group relative to didactic. The pilot nature of this study does not allow sufficient statistical power to conduct a non-inferiority test of group differences on EBPAS change, so comparison of descriptive statistics will provide initial evidence of specificity to be fully tested in a larger trial (61, 62).

The relationship between target change (i.e., reduction in negative beliefs) and outcomes of interest will be evaluated using the EPCS micro-analytic coding system for categorizing in-session exposure delivery behavior (Hypothesis 3). Therapist delivery behaviors during the consultation phase of training will be categorized into “optimal” (i.e., delivery behaviors intended to engage and intensify patient anxiety) and “suboptimal” (i.e., delivery behaviors intended to artificially reduce patient anxiety) categories in accordance with previously established procedures (54). Rates of optimal and suboptimal delivery behaviors will be calculated for each therapist with recorded session data, and in-session behavior will be examined as a function of post-training TBES scores using GLM (Gaussian distribution); these will be conducted as two separate tests examining optimal and suboptimal behaviors. Finally, the relationship between negative beliefs and rates of exposure utilization, calculated as a percentage of sessions with and without exposure delivery, will be assessed using correlational analyses consistent with past training trials incorporating the EPCS coding system [(54, 55); Hypothesis 4].

## Discussion

Despite evidence to support exposure therapy as a frontline treatment for anxiety disorders and OCD it is highly underutilized and often suboptimally delivered in typical practice, even among therapists who have received specialized training in the delivery of exposure therapy (9, 26). A noted barrier to dissemination is therapists’ negative beliefs about the dangerousness and intolerability of exposure therapy for their patients. Preliminary studies have investigated approaches to augmenting negative beliefs during treatment (36, 38, 39), and revealed active strategies such as experiential activities appear to change beliefs above and beyond standard didactic training. The current study aims to apply the same well-validated behavioral strategies used to reduce patient negative beliefs during exposure to target therapists’ negative beliefs and delivery behavior; thus, training was conceptualized as “exposure to exposure.” The study design follows the principles of experimental therapeutics and guidelines put forth by the precision implementation framework for mechanisms of implementation interventions. Findings from the present study will provide a template for applying a rigorous mechanism testing framework on training intervention research, and may help guide the development of targeted training interventions for other EBPs. Due to the pilot nature of this training intervention development project, the sample is not intended to represent a fully powered test of group differences. Instead, the rigorous measurement strategy and planned analyses based on empirical benchmarks provides a strong procedure for determining whether the assumptions of an experimental therapeutics approach are initially met (i.e., target engagement, target-outcome relationship), and can be confirmed in a larger, fully powered training trial.

### Implementation challenges and recommendations

There is strong evidence to support organization-level factors such as implementation climate and transformational leadership as key factors in the successful implementation of evidence-based practices in community settings [e.g., (63, 64)]. However, these organizational factors are not universally supportive of all evidence-based practices and additional research is needed to better understand the unique organization factors that support implementation of exposure therapy (35).

Two significant organization-level factors that influenced the course of this study were changes in partnering clinic sites and significant turnover among both organization leadership and participating therapists.

#### Site changes

Agency 1 provided in-home services at four clinic sites split across two different service divisions. Originally, the study was partnered with two clinic sites led by a division leader that strongly supported training in EBPs and the goals of the current project. Unfortunately, after Phase 1 activities were complete the study team was informed that both partnering clinic sites were closing imminently. The division leader connected the study team to the division leader in charge of the two remaining home-based clinic sites, and the study team quickly pivoted to plan the Phase 2 training trial with a new set of division and program leadership. While many aspects of the programs remained consistent, there were aspects of the supervisor role that differed and influenced study recruitment procedures. Specifically, supervisors at the two new sites were much more involved in case assignments than at our original two sites, and because this was not detected in our rapid onboarding process with the two new sites, supervisors did not receive enough information about identifying and referring relevant study cases to assist with recruitment. This experience underscores the importance of supervisor involvement in successful training implementation efforts (65, 66).

#### Turnover

Initiation of the phase two randomized training trial coincided with the onset of COVID and required a retooling of all planned in-person components to occur remotely (i.e., consenting, measure administration, recruitment, and all training and consultation activities). After piloting and obtaining IRB approval for remote study procedures, the first training was well-attended because therapists were struggling to generate clinical revenue while clinics transitioned to remote care options. Thus, therapists eagerly signed up for the training but had few patients to refer for the consultation component, which contributed to patient recruitment difficulties early in phase two. Relatedly, the “great resignation” (67) occurred during the trial and 57% of trained therapists left their organization during phase two, which again affected patient recruitment rates. Patients were recruited to the study to allow for in-session measures of therapist delivery behavior following training, so patient participants needed to be assigned to a study therapist in order to be eligible for participation. As therapist turnover escalated, the pool of eligible participants shrunk and necessitated an over-recruitment of therapists (36 trained therapists rather than 26 originally proposed) in order for patient recruitment to continue.

Although resources exist to help guide a thorough needs assessment prior to initiating implementation activities in a partnering organization [e.g., (68)], there is much less guidance for implementing therapist training interventions in collaboration with CMHAs. Based on implementation experiences in the current study, it is recommended that early assessment and planning include a thorough understanding of how agency personnel are related hierarchically and with regard to clinical tasks relevant to the study intervention. If there are changes to partnering agencies, sites, or key personnel, as is common when conducting community-based training interventions, it is worth reassessing the organization hierarchy and job duties of all relevant staff to prevent some of the implementation challenges encountered in this study.

### Parallel treatment and E2E training processes

The E2E concept under investigation in the current study provides a template for leveraging existing behavior change strategies validated in clinical practice to move patterns of practice behavior following EBP training interventions. While the present study provides an innovative framework for intervening on therapists’ anxious negative beliefs using experiential training tasks, there are several ways in which exposure principles could be further infused in expanded future tests of the E2E training approach.

#### Assessment

Consistent with assessment of patient symptoms at the outset of treatment, trainers should measure therapist negative beliefs before initiating workshop training to understand the type and extent of therapists’ anxious negative beliefs and tailor didactic and experiential materials accordingly. In the present study beliefs were sampled prior to initiating training to establish a baseline for evaluating change during didactic and experiential portions of the training, but that information was not used to tailor aspects of the workshop. Future iterations of E2E could use baseline beliefs measures to prioritize specific talking points during didactic instruction and focus more time on specific types of experiential tasks. For instance, a therapist endorsing few reservations about the safety of exposure but some concern about patients getting upset during exposure tasks might benefit most from didactic information that presents patient testimonials and experiential tasks in which their partner purposely expresses reservations about an exposure task so the therapist can practice rolling with resistance and titrating the task to be appropriately palatable for the practice partner.

#### Education

Another important parallel process to leverage in future E2E interventions is the link between training didactics and treatment psychoeducation. In both exposure treatment and training, the goal is to ensure individuals understand how and why exposure works to correct anxious beliefs and to set accurate expectations for how to optimize exposure effectiveness. At the outset of treatment, therapists often solicit patient feedback to ensure they understand the concepts and agree with the rationale before proceeding with exposure tasks; future training efforts should include similar learning checks throughout initial didactic components to promote full engagement with ensuing E2E experiential tasks.

#### Hierarchy development

There are many parallel processes between hierarchy development during training and treatment. Hierarchies can consist of both analog and *in-vivo* exposures, and parallel E2E processes for both types of exposure are outlined below.

#### Hierarchy of analog exposures

Experiential tasks during the E2E workshop can be conceptualized as similar to analog exposure tasks with patients. In a course of exposure therapy, patients typically begin with contrived analog exposure tasks (e.g., mock presentation in the office) meant to map on to aspects of situations a person fears as a stepping stone toward eventually approaching naturally occurring feared situations (e.g., presentation at school in front of peers). In the context of exposure treatment, the goal of initial analog hierarchy items is to create individualized opportunities for patients to begin testing their anxious beliefs in proximate situations that allow for a more controlled and gradual approach of feared stimuli. Similarly, the current study utilized individualized fear hierarchies to facilitate self and partner exposures during workshop training, but future efforts may incorporate a wider range of analog training scenarios such as including a third individual playing the role of a parent for therapists who are anxious about parent reactions during exposure delivery.

#### Hierarchy of *in-vivo* exposures

In the context of training, a hierarchy of *in-vivo* exposures represents tasks therapists can attempt in the actual implementation of exposure with their anxious patients. Considerations for gradually bridging the transition from analog (workshop) to *in-vivo* (actual delivery) has received little attention in previous training intervention research; this may be an especially salient barrier to the implementation of exposure following training. The design and evaluation of experiential training tasks that closely represent *in-vivo* delivery, combined with purposeful easing into actual delivery with more straightforward anxiety cases, may be helpful strategies for better bridging the training (workshop) to practice (actual delivery) divide where dissemination so often fails. In the current study, efforts were made to make E2E workshop tasks more representative of feared situations by including partner exposures, but more could be done in future iterations to make delivery feel more representative, such as utilizing immersive virtual reality and delivering exposure with virtual patients. Indeed, it is already challenging to support therapists in identifying candidate patients for exposure delivery following training, but concerted efforts by trainers and supervisors could help therapists start using exposure with less complex case presentations (e.g., simple phobia) before approaching more challenging cases (e.g., complex obsessive–compulsive disorder). By viewing patient presenting characteristics (i.e., symptom complexity, comorbidity, motivation, insight, etc.) and aspects of exposure delivery (i.e., duration, intensity, family involvement, etc.) as potential levers for increasing or decreasing the difficulty of *in-vivo* E2E tasks, there is potential for future training efforts to planfully individualize therapists’ transition from workshop to actual exposure delivery. Strategies that capitalize on this concept may be especially helpful in spanning the well-established dissemination ditch that exists between workshop and actual utilization of exposure in practice.

#### Completing exposure tasks

Consistent with the parallel hierarchy processes above, exposure tasks during both training and treatment can be broken down into analog and *in-vivo* instances.

#### Analog exposures

E2E tasks conducted during workshop offer an opportunity to approach aspects of feared situations (e.g., intensifying exposure with training partner) in a controlled manner before graduating to more realistic exposure scenarios (e.g., intensifying exposure with a patient). Similar to initial exposure tasks during treatment, analog training tasks are as much about targeting and reducing specific anxious beliefs as they are about providing less distressing opportunities to practice exposure procedures and gain confidence in exposure processes before taking on more challenging/realistic exposures in later sessions.

#### *In-vivo* exposures

The implementation of exposure therapy with actual patients during the consultation phase of training can be conceptualized as *in-vivo* E2E. Accordingly, the same principles for optimizing safety learning during treatment [i.e., (58)] are likely to optimize reduction in therapist negative beliefs during *in-vivo* E2E tasks. These principles include: (1) conducting exposure tasks in a prolonged and intense manner, (2) continuing past the point at which individuals expect a feared outcome to occur, (3) repeating exposures across diverse contexts, and (4) reducing the use of safety behaviors. For therapists, safety behaviors might include discontinuing exposures when patients’ anxiety starts to escalate, prescribing the use of coping strategies (e.g., diaphragmatic breathing) during exposure tasks, or providing reassurance (e.g., “No, that spec of dirt is not harmful”) to name a few. Failure to incorporate these principles when structuring therapists’ initial experiences delivering exposure with their patients may inadvertently confirm or exacerbate negative beliefs. For instance, a therapist delivering exposure with low intensity, discontinuing the task as soon as the patient exhibits distress, and apologizing for the patient’s discomfort is likely to foster patient skepticism and hamper the process of symptom reduction. Much like patients, therapists need to experience early success and see that exposure is both tolerable and effective for their patients or they are at risk of discontinuing its use. Future E2E training efforts should focus on leveraging exposure principles during consultation to set up therapists’ initial delivery attempts to maximize the likelihood of disconfirming remaining negative beliefs by leveraging the four exposure principles above.

### Coupling de-implementation with future implementation efforts

Another important process for ensuring successful implementation efforts involves the de-implementation of existing practices. De-implementation is an implicit part of implementation and organizational change that entails substituting a current practice with a related (or unrelated) replacement; the substitution can be either a partial reduction or complete reversal of an existing practice (69). For instance, a training intervention may require educational and technical supports to achieve “unlearning” objectives that effectively steer providers away from outmoded practices. Ideally, de-implementation should be paired with implementation strategies to both steer practitioners away from an existing practice and toward a new practice within the same intervention. Coupling de-implementation and implementation strategies may help reduce confusion about how and when a new practice should be utilized and may also reduce the burden of augmenting or replacing an existing practice. Observations from the current study indicate providers struggled at times to separate the function of specific exposure techniques (e.g., approaching feared stimuli) from techniques presented in a separate prior training (e.g., DBT and ‘grounding’). It is recommended that researchers gather information about other past or ongoing training initiatives at partnering agencies before initiating a new training. Incorporating information about how new training concepts can augment or replace current practices under certain conditions may help with both de-implementation and implementation efforts. Therapists in the current study also struggled at times to find the bandwidth to incorporate a new treatment modality into their typical practice. Future iterations of this training intervention could incorporate strategic de-implementation strategies to get as close to an effort neutral change in practice behavior as possible.

### Summary

This protocol paper describes the rationale for conceptualizing therapists’ negative beliefs about exposure therapy as functionally similar to the anxious beliefs exhibited by patients. Based on this conceptualization, the same exposure principles known to effectively reduce patient anxious beliefs were leveraged to develop targeted experiential tasks intended to reduce therapists’ negative beliefs during training (i.e., conducting training as “exposure to exposure”). Procedures for isolating and evaluating the mechanisms by which the E2E training intervention engages negative beliefs and affects practice behavior were described using the novel precision implementation framework (50). Findings from the present study will provide a template for leveraging change mechanisms with efficacy in a treatment context to enhance the success of training interventions. Future training efforts specific to exposure therapy should prioritize exploration of the parallel processes between exposure treatment and training detailed in the discussion section. Although this study focused on training in a CMHA setting, the training approach is broadly applicable to mental health providers in a variety of settings, including graduate training programs. Future training research in partnership with CMHAs, particularly those involving home-based services, may further benefit from consideration of the implementation challenges and recommendations that resulted from this study.

## Data availability statement

The original contributions presented in the study are included in the article/supplementary material, further inquiries can be directed to the corresponding author.

## Ethics statement

The studies involving human participants were reviewed and approved by Institutional Review Board at Rhode Island Hospital . Written informed consent to participate in this study was provided by the participants’ legal guardian/next of kin.

## Author contributions

There is substantial evidence indicating that therapist negative beliefs about exposure is a therapist-level barrier to its dissemination and optimal delivery. However, existing trainings do not adequately target and reduce this known barrier. The current study involves the novel application of a mechanism-testing framework (i.e., experimental therapeutics) to the design and evaluation of a targeted training intervention for addressing therapist negative beliefs about exposure. The general literature on training therapists in evidence-based practices (EBPs) has consistently demonstrated that passive forms of training like didactic workshops only augment therapists’ knowledge and attitudes about a treatment. The introduction of active (i.e., behavioral) learning strategies is what leads to adherence, competence, and skill change. However, little is known about precisely why the addition of active learning strategies better facilitates behavioral change. The present study is designed to address this critical research gap by testing the effect of novel behavioral training strategies on a target (i.e., therapist beliefs) related to our behavioral outcome of interest (i.e., quality exposure delivery). All authors listed have made a substantial, direct, and intellectual contribution to the work and approved it for publication.

## Funding

Research activities pertaining to this manuscript were supported by funding from the National Institute of Mental Health (grant# 1R34MH118199).

## Conflict of interest

The authors declare that the research was conducted in the absence of any commercial or financial relationships that could be construed as a potential conflict of interest.

## Publisher’s note

All claims expressed in this article are solely those of the authors and do not necessarily represent those of their affiliated organizations, or those of the publisher, the editors and the reviewers. Any product that may be evaluated in this article, or claim that may be made by its manufacturer, is not guaranteed or endorsed by the publisher.
